# P-1839. Emergency Department-Based Opt-out Screening and Linkage to Care for Hepatitis C Virus within a Community Healthcare System

**DOI:** 10.1093/ofid/ofaf695.2008

**Published:** 2026-01-11

**Authors:** Paula A Eckardt, Jianli Niu, Sheila Montalvo

**Affiliations:** Memorial Healthcare System, Hollywood, FL; Memorial Healthcare System, Hollywood, FL; Memorial Hospital System, Cooper City, FL

## Abstract

**Background:**

Hepatitis C virus (HCV) remains a significant public health challenge in the United States. Our institute implements HCV screening and linkage-to-care services through Gilead’s Frontlines of Communities in the United States (FOCUS) program. We aimed to report our early experience with emergency departments (ED)-based “Opt-out” HCV screening and care linkage program within a community healthcare setting in South Florida.

**Figure 1.**
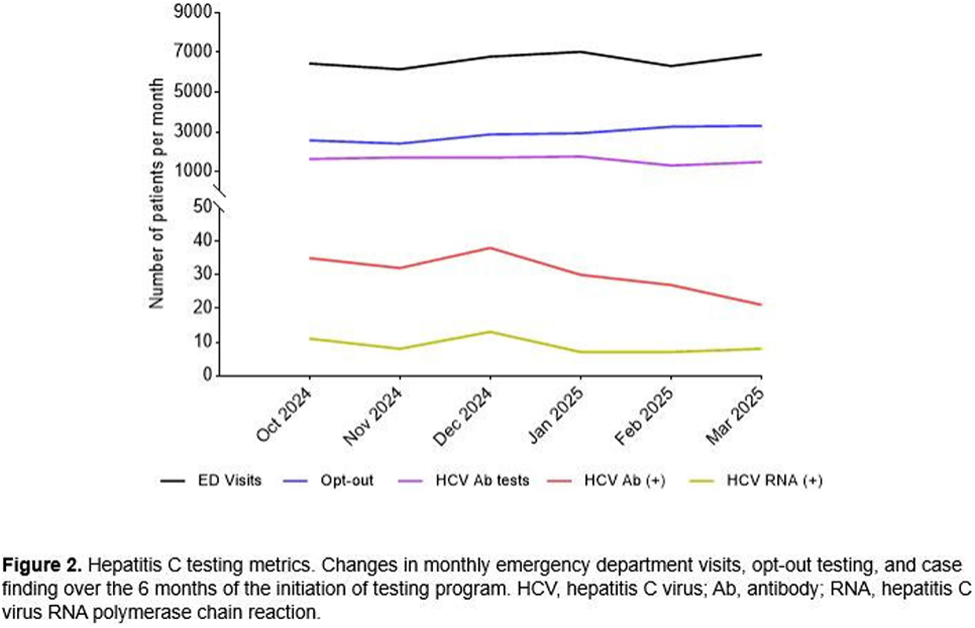
HCV testing metrics-changes in monthly ED visits, opt-out testing, and case findings over 6 months or program initiation

**Methods:**

We implemented “Opt-out” HCV screening program to patients aged 18 years or older presenting to the ED and offered linkage to care to patients with a positive result for HCV on an RNA test. Data from October 2024 to March 2025 on ED visits, HCV testing offered, number of patients screened, tested reactive for HCV antibody, test reactive for HCV RNA, and linkage to care were analyzed.

**Results:**

Figure 1 presents HCV testing metrics and illustrates monthly trends in testing volume and outcomes. During a 6-month period, 22,283 (56.3%) patients of 39,268 ED visits were offered for HCV testing and 9,565 (42.9%) completed HCV testing. Of those tested, 177 (1.9%) patients tested positive for HCV antibody, with 54 (30.5%) of whom were positive for HCV RNA. Of these 54 patients, 61.1% were male, 57.4% were non-Hispanic White, followed by Hispanic (22.2%), non-Hispanic Black (18.5%), and Asian/Pacific Islander (1.9%) persons. 21(38.9%) were newly diagnosed for HCV infection and 33 (61.1%) were known for HCV who had not disclosed their status. The median age of the newly diagnosed patients was 44 (interquartile range [IQR], 37−49) years versus 43 (IQR, 39−57) years for known infected patients (p = 0.569). 9 (42.9%) of 21 newly diagnosed patients were successfully linked to care. 11 (33.3%) of 33 patients with known HIV infections who were not engaged in HCV care were successfully relinked into care after testing, and engagement in care increased from 12.1% pre-testing to 45.5% post-testing (p = 0.006).

**Conclusion:**

ED-based HCV screening and linkage-to-care services play a pivotal role in identifying new HCV cases and enhancing care linkage outcomes.

**Disclosures:**

All Authors: No reported disclosures

